# Beyond the WHO Priority Toxicants: A Systematic Review of Harmful and Potentially Harmful Constituents in IQOS Aerosols

**DOI:** 10.3390/toxics14070614

**Published:** 2026-07-13

**Authors:** Roxana Ioana Matei, Anda Maria Baroi, Toma Fistos, Irina Fierascu, Radu Claudiu Fierascu

**Affiliations:** 1 National Institute for Research & Development in Chemistry and Petrochemistry—ICECHIM Bucharest, 202 Splaiul Independenței, 060021 Bucharest, Romania; roxana.brazdis@icechim.ro (R.I.M.); anda.baroi@icechim.ro (A.M.B.); toma.fistos@icechim.ro (T.F.); irina.fierascu@icechim.ro (I.F.); 2 Faculty of Horticulture, University of Agronomic Sciences and Veterinary Medicine of Bucharest, 59 Marasti Blvd, District 1, 011464 Bucharest, Romania; 3 Faculty of Chemical Engineering and Biotechnologies, National University of Science and Technology Politehnica Bucharest, 1-7 Gheorghe Polizu St., 011061 Bucharest, Romania; 4 Academy of Romanian Scientists, 3 Ilfov Str., 050044 Bucharest, Romania

**Keywords:** heated tobacco products, HPHCs, aerosol chemistry, toxicants

## Abstract

Heated tobacco products (HTPs) generate an inhalable aerosol by heating tobacco instead of burning it as conventional cigarettes do. Although several harmful and potentially harmful constituents (HPHCs) are generally reduced compared with conventional cigarette smoke, the World Health Organization’s list of nine priority toxicants (WHO 9) may not fully reflect the chemical complexity of HTP aerosols. The main goal of this review is to evaluate the occurrence, quantitative levels, and toxicological relevance of HPHCs identified in IQOS aerosols and to propose an expanded toxicant panel for aerosol characterization. The review was conducted according to PRISMA 2020 recommendations using literature retrieved from the Web of Science Core Collection database. The studies included in the present review consistently report lower levels of many combustion-related toxicants in IQOS aerosols, compared with conventional cigarette smoke, particularly for carbon monoxide, volatile organic compounds, and polycyclic aromatic hydrocarbons. However, several toxicologically relevant compounds, including carbonyls, tobacco-specific nitrosamines, phenolics, and trace metals, remained detectable in several studies. An expanded panel of twenty priority toxicants was proposed, based on toxicological relevance, frequency of detection, regulatory significance, and analytical feasibility. The proposed panel extends beyond the WHO 9 framework while preserving regulatory applicability and analytical practicality. The findings support the need for harmonized methodologies and broader toxicant monitoring strategies for the comparative assessment of heated tobacco products.

## 1. Introduction

Tobacco smoking is among the primary causes of disease and early mortality worldwide and is strongly linked to cardiovascular illnesses, respiratory disease, and multiple types of cancer [1,2]. While nicotine constitutes the compound primarily responsible for addiction, smoking-related adverse health effects are related to chronic exposure to Harmful and Potentially Harmful Constituents (HPHCs) formed in the process of tobacco combustion [3,4,5,6,7]. Mainstream cigarette smoke consists of thousands of compounds in particulate and vapor phases, including carbonyls, volatile organic compounds (VOCs), polycyclic aromatic hydrocarbons (PAHs), tobacco-specific nitrosamines (TSNAs), phenolics, and toxic metals [3,4]. Many of these substances are involved in carcinogenesis, oxidative stress, inflammation, endothelial dysfunction, and respiratory toxicity [5,6,7,8,9,10,11], forming the basis of tobacco emission characterization that remains critical for tobacco product analysis and regulation. In this regard, there has been an increased interest in alternative products of nicotine delivery aimed at modifying the chemical composition of the generated aerosol by avoiding tobacco combustion.

Heated tobacco products (HTPs) generate an aerosol through the controlled heating of processed tobacco instead of combustion. While conventional cigarettes reach peak temperature above 800 °C during puffing, HTPs typically operate at substantially lower temperatures (<350 °C), thus reducing the measured levels of many combustion-related toxicants in the generated aerosol [12,13,14,15]. However, even though HTPs are less likely to produce such compounds as a consequence of sustained combustion, other toxicants are still generated, including nicotine, carbonyls, volatile organic compounds, tobacco-specific nitrosamines, particulate matter, and thermal degradants [16,17]. Thus, HTPs need to be considered as a separate class of tobacco products for toxicant profiling.

IQOS, a commercially available heated tobacco system (HTS) developed by Philip Morris International (PMI) that generates an aerosol by heating processed tobacco instead of burning it, is one of the most extensively studied heated tobacco products and therefore represents one of the primary subjects of analytical and toxicological investigations [12]. Whereas earlier IQOS generations employed electrically heated blades to heat tobacco sticks, more recent ILUMA devices heat tobacco through an induction-heating process involving metal susceptors embedded in consumables [18]. IQOS aerosol is formed through evaporation, distillation, condensation, and low-temperature thermal degradation, processes that occurs without sustained combustion [18]. Nevertheless, several studies have reported the presence of products of pyrolytic transformation in IQOS aerosol, implying that even lower operating temperature does not entirely exclude thermal degradation reactions [19]; moreover, under certain puffing parameters, nicotine yield from IQOS approaches cigarette-like levels [20,21].

Current approaches to tobacco toxicant monitoring are primarily based on regulatory frameworks, such as WHO 9 priority toxicants and FDA (U.S. Food and Drug Administration) HPHCs list [6,7]. Although these approaches are highly relevant and useful, they are mainly applicable to combustible cigarette smoke and might be somewhat incomplete when used to characterize heated tobacco aerosols generated under different conditions. On the other hand, comparison between different studies is hampered by the variation in puffing regimens, analytical approach, generation of devices and consumables used, and units of quantification [22,23,24].

WHO 9 priority toxicants comprise three carbonyls (formaldehyde, acetaldehyde, and acrolein), two VOCs (benzene and 1,3-butadiene), carbon monoxide, benzo[a]pyrene, and two TSNA: NNK ((4-(methylnitrosamino)-1-(3-pyridyl)-1-butanone) and NNN ((N′-nitrosonornicotine)) [6]. This set of chemicals constitutes an important tool for the assessing toxicological impact of smoking-related toxicants on human health and has been proven to be a reliable framework for tobacco toxicant analysis. However, a growing body of evidence suggests that HTPs generate not only WHO 9 chemicals, but also other toxicants such as carbonyl products derived from humectants, semivolatile compounds, flavor degradants, phenolics, and trace metals, which could not be included in the classical WHO framework [9,22,23,24].

Considering the abundance of available information and the presence of both producer-affiliated and independent analyses, IQOS aerosol is a good platform for chemical assessment of heated tobacco aerosols. Furthermore, IQOS has been subject to extensive analysis in relation to regulatory assessment as modified risk tobacco product by FDA [25].

This review systematically examines the occurrence, quantitative levels, toxicological relevance, and analytical determination of harmful and potentially harmful constituents (HPHCs) reported in IQOS aerosols. Beyond synthesizing the available evidence, it proposes an expanded panel of priority toxicants selected according to four complementary criteria: toxicological relevance, frequency of detection across the literature, regulatory significance, and analytical feasibility. Together, these criteria provide a transparent basis for prioritizing toxicants for aerosol monitoring. The resulting panel complements the WHO 9 framework by offering a practical tool for more comprehensive aerosol characterization while remaining suitable for routine analytical investigations and future regulatory applications.

## 2. Methodology

### 2.1. Search Strategy

This systematic review was conducted following the Preferred Reporting Items for Systematic Reviews and Meta-Analyses (PRISMA 2020) recommendations [26]. The review strategy was developed using the PICO (Problem, Intervention, Comparison, Outcome) framework presented in Table 1.

The literature search was conducted on 30 January 2026, using the Web of Science Core Collection database. Although additional databases such as Scopus and PubMed may contain relevant studies, Web of Science Core Collection was selected because of its extensive coverage of the peer-reviewed literature and its suitability for citation-based screening. No publication year restrictions were initially applied in order to capture the full range of available studies related to heated tobacco aerosol characterization.

The purpose of the search strategy was to detect studies describing aerosols generated by heated tobacco products and their chemical composition, in particular, IQOS devices and their consumables. The search keywords were: “Tobacco Heating System”, “THS”, “heated tobacco product”, “modified risk tobacco” and “heat-not-burn”. Refining keywords included: “aerosol”, “composition”, “compound”, and “determination”.

During the screening, editorials, meeting abstracts, reviews, notes, data papers and proceedings without description of methodology were excluded, as well as papers published in languages other than English.

### 2.2. Eligibility Criteria

Studies were considered eligible for further analysis if they met the following criteria:

(i) The article was indexed in the Web of Science Core Collection database;
(ii) The study investigated IQOS devices or IQOS-compatible consumables;
(iii) The work included experimental or analytical characterization of aerosol composition;
(iv) Aerosol generation was performed under controlled laboratory conditions using clearly described puffing regimens or smoking-machine protocols;
(v) The study reported quantitative or semi-quantitative data for harmful and potentially harmful constituents (HPHCs);
(vi) Sufficient methodological information regarding aerosol collection and analytical determination was provided.

Studies concentrated on consumer perceptions, marketing and behavioral analysis, or biomarkers only were excluded. Also excluded were those investigations focused on other heated tobacco products not relevant to IQOS devices.

### 2.3. Study Selection

Duplicate records were excluded before proceeding to titles and abstracts screening independently performed by two authors. Each title/abstract was assigned into three categories: “include”, “exclude” or “uncertain”. Papers, categorized as “include” or “uncertain” at this first screening step, proceeded to the next one where a full-text examination was performed.

**Figure 1:**
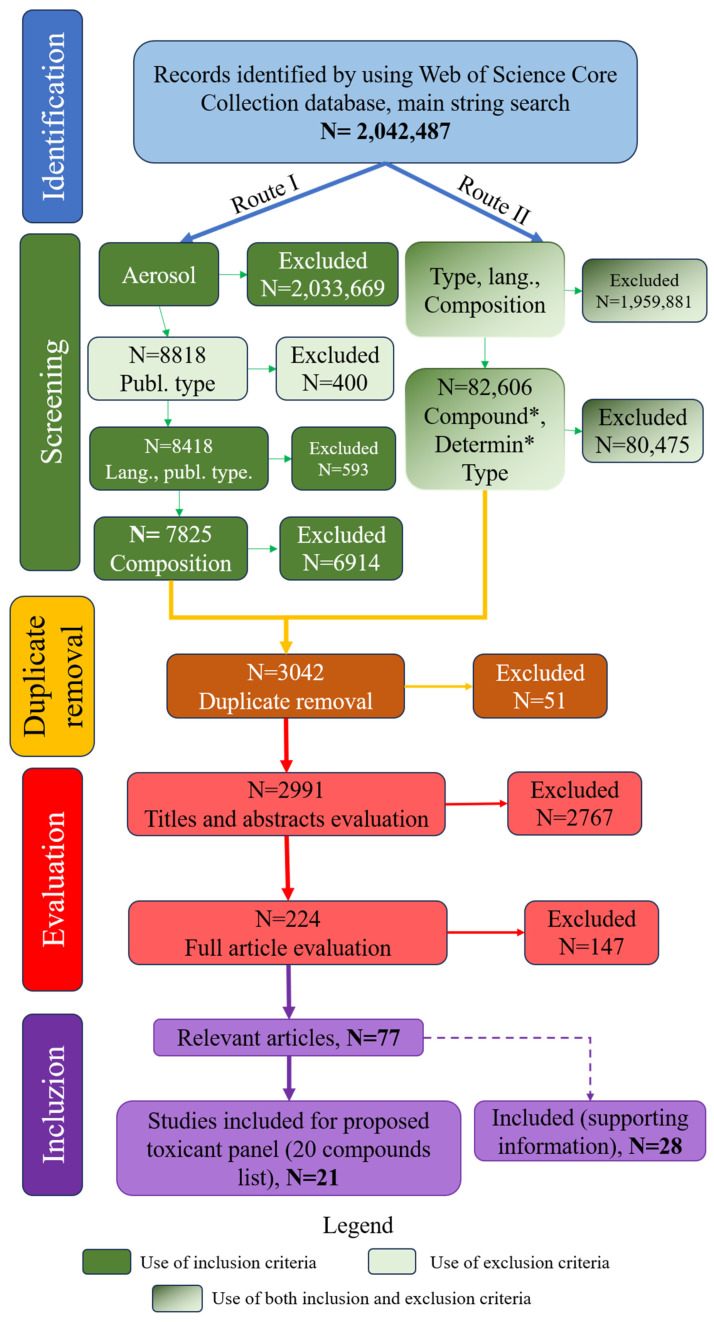
PRISMA-based workflow describing study identification, screening, eligibility assessment, and selection for systematic synthesis.

Any disagreements about inclusion/exclusion decisions were discussed until agreement was reached. The study selection process is summarized in Figure 1, while the complete PRISMA 2020 flow diagram is provided in the Supplementary Materials.

### 2.4. Data Extraction

Data extraction was independently performed by two reviewers using a standardized collection framework. For each included study, the following information was recorded:

➢ bibliographic information;
➢ study type and primary objective;
➢ IQOS device generation;
➢ consumable type;
➢ puffing regimen;
➢ comparator cigarette;
➢ aerosol matrix analyzed;
➢ target analytes;
➢ analytical methods;
➢ reporting units;
➢ analytical quality parameters, when available;
➢ institutional affiliation, funding source, and declared conflicts of interest, when reported.

Particular attention should be paid to puffing regimens, aerosol collection systems, analytical sensitivity and units used for reporting data, since they are critical points in comparing quantitative data between studies.

To improve the transparency of the systematic review process, detailed summaries of the publications considered during evidence synthesis are provided as Supplementary Materials. Supplementary Table S1 presents the characteristics of the analytical studies that fulfilled the predefined eligibility criteria and were used to develop the proposed toxicant panel, whereas Supplementary Table S2 summarizes the additional publications consulted to support the interpretation of toxicological relevance, analytical methodologies, and regulatory context. Besides these works, a series of other studies (regulatory materials and review-type studies were directly retrieved from the database).

### 2.5. Methodological Considerations and Limitations

Several methodological limitations should be considered when interpreting the inputs of the present review. First, the analysis was based on studies indexed in the Web of Science Core Collection database, meaning that relevant studies indexed exclusively in other databases may not have been captured.

In addition, substantial heterogeneity over the available literature regarding puffing regimens, device generations, aerosol collection systems, analytical methodologies, and reporting units was observed. These differences complicate direct quantitative comparison between studies and may contribute to variability in reported toxicant levels. Because of the substantial heterogeneity across the literature, a formal quantitative meta-analysis was not considered methodologically appropriate.

The evidence supporting the proposed toxicant panel was derived exclusively from the studies that fulfilled the predefined eligibility criteria for the systematic review. Additional publications were consulted to provide supporting information on toxicological mechanisms or analytical methodologies, but were not used to determine the inclusion of compounds in the proposed panel. Furthermore, the available evidence includes both independent and industry-associated studies. Rather than assigning greater weight to studies based on institutional affiliation, the interpretation of the evidence relied on the overall consistency of the reported findings, taking into account methodological transparency, analytical quality, and the declaration of funding sources and potential conflicts of interest.

Another important consideration is the rapid evolution of heated tobacco technologies. A large proportion of the currently available literature focuses on earlier IQOS generations, particularly THS 2.2, while newer systems such as ILUMA remain less extensively characterized.

The present review did not apply a formal risk-of-bias scoring tool as the included studies were analytical chemistry investigations and not clinical or epidemiological studies, for which such tools were originally developed. Instead, the evidence was interpreted by considering methodological transparency, analytical detail, reproducibility, and the declaration of funding source, institutional affiliation (producer-affiliated or independent), and declared conflicts of interest. The distinction between studies included in the systematic synthesis and publications used as supporting evidence is presented in Supplementary Tables S1 and S2, respectively.

## 3. Framework for Aerosol Characterization and Toxicant Selection

### 3.1. Technological and Physicochemical Aspects of IQOS Aerosol

Unlike conventional cigarettes, where temperatures in the combustion zone can reach 800–900 °C, IQOS heats tobacco without sustaining complete combustion through evaporation, distillation, and condensation processes that occur without reaching excessively high temperatures (>350 °C) [3]. While the earlier versions of IQOS (such as THS 2.2), used electrically heated metal blades, the recent generations (such as ILUMA) use induction heating with an embedded metallic susceptor [15,22].

IQOS aerosol differs considerably from conventional cigarette smoke regarding the physical composition, being mostly constituted of semi-volatile liquid droplets containing water, glycerol, and dissolved nicotine and other organic compounds [27,28,29]. Glycerol serves as an aerosol former and humectant; however, upon heating, it decomposes to produce several compounds, such as formaldehyde, acrolein, and acetaldehyde [27,30]. Although many of combustion-derived compounds are present in smaller quantities in IQOS aerosol, reduced-temperature operation does not eliminate the possibility of thermal reaction leading to production of several other compounds.

Several studies have reported the presence of aldehydes, VOCs, TSNAs, and markers of pyrolytic decomposition in IQOS aerosol [18,19,31]. While certain toxicants originate from tobacco matrices, other compounds are produced as a result of thermal reaction involving the decomposition of glycerol, sugars, cellulose, and other tobacco components [30,31].

Comparison of results across the various analytical studies is made extremely difficult due to dependence of aerosol composition on puffing conditions, specific devices used, consumables characteristics, and analytical method employed. Most common puffing regimens used in IQOS aerosol investigation are the ISO and Health Canada Intense (HCI) puffing regime [32,33,34,35,36].

Semi-volatility of IQOS aerosol poses unique analytical challenges for toxicant monitoring. Various aerosol sampling methods have been described in the literature, ranging from simple Cambridge filter pads up to complex online interfaces, electrostatic aerosol traps, impingers, and gas bags [37,38]. Analytical methods commonly include spectrometry-based and chromatography-based assays, such as GC-MS, LC-MS/MS, HPLC, and ICP-MS [37,38]. Consequently, the literature analysis requires taking aerosol sampling methodology and analytical sensitivity, puffing regimens, and device generation into account.

### 3.2. Regulatory and Scientific Basis for Toxicant Selection

The current approach to monitoring of tobacco products relies on toxicant sets that were initially developed for cigarette smoke. WHO framework includes nine priority toxicants, whereas U.S. Food and Drug Administration list comprises harmful and potentially harmful constituents (HPHCs) [6,7]. Both panels are valuable, being focused on compounds having strong toxicological properties.

The WHO 9 panel includes three carbonyls (formaldehyde, acetaldehyde, and acrolein), two VOCs (benzene and 1,3-butadiene), carbon monoxide, benzo[a]pyrene, and two TSNAs: NNK ((4-(methylnitrosamino)-1-(3-pyridyl)-1-butanone) and NNN (N′-nitrosonornicotine) [6]. Despite its practical utility in terms of standardized analytical protocol development, WHO 9 panel is mainly relevant for the characterization of conventional cigarette smoke and it cannot be applied to heated tobacco products in a straightforward way [12,22,39].

Whereas many combustion-related compounds have generally been reported at lower measured concentrations in IQOS aerosol compared to cigarette smoke, IQOS aerosol also contains significant amounts of compounds that cannot be considered as combustion-related products [18,23,29]. Therefore, the WHO 9 panel, limited to specific class of chemical compounds, is not able to reflect the entire chemical diversity of heated tobacco aerosols.

HPHCs panel includes a more diverse range of substances, including carbonyls, VOCs, PAHs, TSNAs, phenolics, aromatic amines, toxic gases, and metals [7]. Although HPHCs panel is much more representative, not all the HPHCs are relevant for IQOS aerosols and vice versa. In addition, HTPs often contain substantial amount of other chemicals not included in classical cigarette-oriented toxicant frameworks.

To improve the transparency of toxicant selection, the proposed panel was established using a structured four-criterion framework. Each candidate toxicant was evaluated according to (i) toxicological relevance, considering evidence of carcinogenicity, respiratory, cardiovascular, or systemic toxicity [6,9,14,29,39]; (ii) frequency of detection across the reviewed literature, prioritizing compounds consistently reported by multiple independent investigations; (iii) regulatory significance, including compounds already incorporated into internationally recognized monitoring frameworks; and (iv) analytical feasibility, favoring analytes that can be reliably quantified using standardized laboratory methods (emphasis was placed on compounds that could be analyzed by GC-MS, LC-MS/MS, HPLC, and ICP-MS analyses). Only compounds consistently reported across multiple eligible studies were considered for inclusion in the proposed panel, thereby reducing the influence of isolated observations.

The evidence supporting the proposed panel was derived from the 21 studies that fulfilled the predefined eligibility criteria for inclusion in the systematic review. Of these, 11 were conducted by independent research groups (out of which 2 studies were financed by Philip Morris International), eight were performed exclusively by researchers affiliated with the IQOS manufacturer (Philip Morris International), one involved researchers affiliated with another tobacco manufacturer, and one represented a collaboration between industry-affiliated and independent authors. Additional publications were consulted to provide supporting information on toxicological relevance, analytical methodologies, and regulatory aspects, but they were not used for toxicant selection. This distribution illustrates the diversity of the evidence base considered in the present review and provides additional context for interpreting the proposed toxicant panel.

Unlike previous works, which primarily summarize the occurrence of HPHCs or compare emission levels between heated tobacco products and conventional cigarettes, the present review focuses on the systematic selection of a practical toxicant panel for IQOS aerosol characterization. Rather than compiling an exhaustive list of reported compounds, our objective was to identify analytes that are simultaneously toxicologically relevant, consistently detected across independent studies, recognized within current regulatory frameworks, and amenable to standardized analytical determination. This approach seeks to bridge the gap between descriptive evidence and the practical requirements of routine laboratory monitoring and regulatory assessment. Based on these predefined selection criteria, an expanded panel of twenty priority toxicants for IQOS aerosol assessment was compiled (Table 2). The proposed panel (derived from the subset of studies that reported comprehensive analytical datasets covering the principal classes of HPHCs and met the predefined selection criteria described above) preserves continuity with internationally recognized toxicants while providing broader coverage of compounds relevant to heated tobacco aerosol chemistry, particularly reactive carbonyls, VOCs, phenolics, and trace metals.

## 4. Occurrence of Harmful and Potentially Harmful Constituents in IQOS Aerosols

Quantitative assessment of harmful and potentially harmful constituents (HPHCs) in IQOS aerosols is essential for understanding exposure patterns and comparing heated tobacco emissions with conventional cigarette smoke. However, direct comparison across studies remains difficult because toxicant levels are reported using different units, smoking regimens, device generations, and analytical approaches [13,40,41].

Most studies present the results of aerosol composition as μg/stick, ng/stick, μg/puff, or μg/session, while some investigations report concentrations in diluted aerosol systems or exposure chambers [22,33,41]. In addition, aerosol composition may vary depending on puffing conditions, particularly between ISO and Health Canada Intense (HCI) regimens [34,35]. In general, more intensive puffing increases aerosol mass and significantly influences the measured levels of carbonyl compounds, VOCs, and other thermally generated constituents [22,42].

Another important source of variability is represented by differences between IQOS generations, consumable formulations, aerosol trapping strategies, and analytical sensitivity. Several compounds are present at trace levels close to detection or quantification limits, meaning that relatively small methodological differences may lead to substantial variations between studies. Consequently, quantitative findings should always be interpreted in the context of the applied methodology, reporting units, and smoking protocol.

Table 3 summarizes the concentrations of major HPHCs reported in IQOS aerosols and comparator conventional or standard cigarette smoke reported on the studies included in the present review (units were retained as reported in the original studies to preserve methodological consistency and avoid introducing conversion-related uncertainties).

### 4.1. Carbonyl Compounds

The following section summarizes the analytical findings reported in the eligible studies regarding carbonyl compounds. Their relevance for toxicant selection is discussed in Section 5.

Carbonyl compounds are among the most consistently reported toxicants in IQOS aerosols and are primarily formed through thermal degradation of glycerol, sugars, and other tobacco constituents during heating [8,28]. The most frequently investigated compounds include formaldehyde, acetaldehyde, acrolein, crotonaldehyde, and propionaldehyde.

Both producer-affiliated and independent studies consistently report significantly lower measured carbonyl levels in IQOS aerosols under the investigated experimental conditions, compared with conventional cigarette smoke, although the reduction level varies depending on the compound, puffing regime, as well as the analytical methodology used. Schaller et al. reported reductions of 80–95% for formaldehyde, acetaldehyde, and acrolein, compared to the 3R4F reference cigarette, under the Health Canada Intense regime [3,14]. Similar trends were also confirmed by independent investigations, including studies by Mallock et al. and Bekki et al. [4,41].

Despite these lower levels, carbonyl compounds remain toxicologically relevant because several aldehydes are highly reactive and strongly associated with oxidative stress, respiratory irritation, and epithelial injury [31]. Acrolein is of particular interest due to its pronounced electrophilic character and its potential contribution to inflammatory and cardiovascular effects. In addition, more intensive puffing conditions may increase carbonyl formation, thus supporting the importance of smoking regimen standardization when comparing quantitative results from different studies [13,40,41,42].

### 4.2. Volatile Organic Compounds (VOCs)

Volatile organic compounds such as benzene, 1,3-butadiene, acrylonitrile, toluene, and styrene are important markers of combustion-related toxicity in tobacco emissions. In IQOS aerosols, these compounds are usually detected at significantly lower measured concentrations than in conventional cigarette smoke, a consequence of the lower operating temperatures and of the absence of combustion processes [4,31,32].

Most of the studies report reductions exceeding 90% for benzene and 1,3-butadiene comparative to conventional cigarettes [3,31]. Similar trends have been observed for acrylonitrile and toluene regardless the type of the study (industry-affiliated and independent studies) [4,43]. Nevertheless, measurable concentrations of several VOCs remain detectable in IQOS aerosols, indicating that thermal degradation and transfer processes still contribute to VOC formation under heated tobacco conditions.

As is the case for other classes of compounds, interpretation of the data presented for VOC analysis is also challenging, as the analyte levels are strongly influenced by puffing intensity, aerosol collection methods, and analytical sensitivity. Some compounds are present near detection limits, contributing to variability across studies. Even so, the available literature consistently reports substantially lower measured VOC levels in IQOS aerosol than in conventional cigarette smoke [3,4,44].

### 4.3. Toxic Gases

The following section summarizes the analytical findings reported in the eligible studies regarding toxic gases. Their relevance for toxicant selection is discussed in Section 5.

Among gaseous toxicants, carbon monoxide (CO) remains one of the most relevant markers of combustion-related emissions. Because IQOS heats tobacco instead of burning it, CO levels are consistently lower than those reported for conventional cigarette smoke [14,31,45]. Most studies included in the present review reported reductions in CO levels of up to 95%, with several investigations reporting CO concentrations close to or below the limit of quantification under standard puffing conditions [15,31].

The substantially lower CO concentrations reported across studies are consistent with the lower operating temperatures of heated tobacco systems and the absence of sustained combustion. Nevertheless, measurable CO concentrations have still been reported in some studies, particularly under more intensive puffing conditions or depending on the analytical methodology used [46]. Presented results suggest that limited thermal decomposition and incomplete oxidation reactions may still occur during aerosol generation.

Other gaseous compounds, including ammonia and hydrogen cyanide (HCN), have been investigated less frequently, most probably as their precise quantification requires more specialized sampling and analytical approaches [45,46]. Available studies generally report lower concentrations than in cigarette smoke, although methodological variability makes direct comparison difficult, like for other compounds. Even at reduced levels, these compounds remain relevant because of their potential contribution to respiratory irritation and systemic toxicity.

Overall, the analytical studies consistently report lower measured concentrations of combustion-related gaseous toxicants in IQOS aerosol than in conventional cigarette smoke. However, the same parameters such as differences in puffing regimens, trapping systems, and analytical sensitivity continue to represent important sources of variability in all the studies.

### 4.4. Tobacco-Specific Nitrosamines (TSNAs)

The following section summarizes the analytical findings reported in the eligible studies regarding tobacco-specific nitrosamines. Their relevance for toxicant selection is discussed in Section 5.

Tobacco-specific nitrosamines (TSNAs), particularly NNK and NNN, are among the most important carcinogenic constituents associated with tobacco use. Unlike many combustion-derived toxicants, TSNAs are primarily formed during tobacco curing and processing and are subsequently transferred from the tobacco matrix into the aerosol during product use [8,47]. As a result, reductions in TSNA levels in IQOS aerosols are generally less pronounced than those observed for other compounds, directly linked to combustion [48].

Both industry-affiliated and independent studies consistently report detectable levels of NNK and NNN in IQOS aerosols, typically in the ng/stick range [8,31,32]. Compared with conventional cigarette smoke, reductions range between 70% and 97%, depending on the study design, smoking regime, as well as the analytical method used [14,32,49]. Similar trends have been observed across multiple IQOS generations and consumable types.

The continued detection of TSNAs is particularly important from a toxicological perspective because these compounds are strongly associated with carcinogenic mechanisms involving metabolic activation and DNA adduct formation [47]. Their persistence in heated tobacco aerosols highlights one of the key differences between combustion-derived toxicants and constituents inherently associated with the tobacco material itself.

Despite the quantitative differences observed between different studies, the available evidence consistently supports the inclusion of TSNAs among the priority analytes for IQOS aerosol characterization. Their relatively stable presence reported in different analytical investigations also makes them useful comparative markers for evaluating heated tobacco emissions.

### 4.5. Polycyclic Aromatic Hydrocarbons (PAHs)

The following section summarizes the analytical findings reported in the eligible studies regarding polycyclic aromatic hydrocarbons. Their relevance for toxicant selection is discussed in Section 5.

Polycyclic aromatic hydrocarbons (PAHs) are classical combustion-related toxicants formed mainly during incomplete burning of organic material. As IQOS-type devices operates at significantly lower temperatures than conventional cigarettes, PAH levels in its aerosol are generally very low and often close to analytical detection limits [4,32].

Benzo[a]pyrene is the most frequently monitored PAH because of its carcinogenic potential and its widespread use as a regulatory marker [50]. In the studies included in the present review, benzo[a]pyrene concentrations in IQOS aerosols were reported at trace levels and substantially below those measured in cigarette smoke [3,30,31]. Similar findings were observed for other PAHs, such as naphthalene, although fewer studies investigated extended PAH panels.

One of the clearest analytical differences between IQOS aerosols and conventional cigarette smoke is the consistently lower measured PAH concentrations. Nevertheless, trace detection of PAHs in several studies suggests that limited pyrolytic reactions may still occur during heating, particularly under intensive puffing conditions or localized overheating events [18].

Interpretation of PAH data should also consider the analytical difficulty associated with measuring compounds present at extremely low concentrations. Small methodological differences in aerosol collection, extraction efficiency, and instrumental sensitivity may therefore contribute to inter-study variability. Despite these limitations, the overall literature consistently reports substantially lower measured PAH concentrations in IQOS aerosol compared with combustible cigarettes.

### 4.6. Toxic Metals

The following section summarizes the analytical findings reported in the eligible studies regarding toxic metals. Their relevance for toxicant selection is discussed in Section 5.

Toxic metals represent a distinct class of aerosol constituents because, unlike organic compounds, they are not generated through combustion or thermal degradation reactions; metals may originate from the tobacco material itself, device components, or contamination introduced during manufacturing and processing [49].

The metals most frequently investigated in IQOS aerosols include cadmium, lead, nickel, and mercury. Reported concentrations are generally low and often close to detection or quantification limits, although variability between studies remains considerable [32,49]. Several investigations reported cadmium and lead levels below the limit of quantification in IQOS aerosols, while others detected trace concentrations depending on the analytical sensitivity and aerosol collection strategy employed.

Compared with many organic toxicants, interpretation of metal data is more complex because toxicity depends not only on total concentration, but also on chemical speciation, particle size, bioavailability, and long-term accumulation potential [51,52,53]. In addition, the relatively low concentrations reported in heated tobacco aerosols make accurate quantification analytically challenging.

Although metals are generally present at much lower levels than many major organic toxicants, their inclusion in aerosol assessment remains important, as chronic exposure to cadmium, lead, nickel, and mercury has been associated with cardiovascular, neurological, respiratory, and carcinogenic effects [51]. Their persistence across different tobacco and aerosol systems also supports their use as complementary markers in comprehensive toxicant monitoring strategies.

Taken together, the analytical studies included in this review consistently report substantially lower measured concentrations of many combustion-related toxicants in IQOS aerosols than in conventional cigarette smoke. The largest decreases are generally observed for carbon monoxide, PAHs, and several volatile organic compounds, while smaller decreases are reported for compounds more closely associated with the tobacco matrix itself, such as TSNAs. However, considerable variability remains across studies because aerosol composition is strongly influenced by device generation, puffing conditions, aerosol collection methods, analytical sensitivity, and reporting units. These differences highlight the need for harmonized methodologies and carefully selected toxicant panels when comparing heated tobacco products across studies and regulatory frameworks.

**Table 3:** Concentration of HCHPs determined in HTP aerosols compared to conventional/standard cigarette smoke. The values described as HTP_1_ or HTP_2_ correspond to different determination conditions (presented in table). Type of study is presented as PMI (published by authors affiliated with the producer-Philip Morris International), respectively Independent (published by authors not affiliated with PMI); ND—not detected; BDL, <LOD—values under detection limit, <LOQ—values under the limit of quantification; % reduction is only provided when presented by the authors.

Compound	Type of Study	Reported Unit	Value			% Reduction HTP vs. Comparator Cigarette (Reported by Authors)	Ref.
			HTP_1_	HTP_2_	Comparator Cigarette		
**Carbonyls**							
Acetaldehyde	PMI	μg/stick	219 ± 31/213 ± 19 (regular)	205 ± 12/220 ± 22 (menthol)	1555 ± 184/1589 ± 76	-	[3]
		μg/unit	230 ± 21 (in synthetic air)	211 ± 16 (in nitrogen)	1656 ± 26	-	[15]
		ng/article	217 ± 7.85	-	1641 ± 258	86.8	[31]
		μg/stick	197.2 ± 15.6 (regular)	199.4 ± 13.5 (menthol)	1713 ± 123	88.49–88.36	[32]
		μg/item	166 ± 5.44 (regular)	191 ± 6.21 (menthol)	1574 ± 106	89.5–87.9	[49]
		μg/stick	219 ± 10	-	1555 ± 38	86	[52]
		μg/mL	1.58	-	21.4 (Marlboro Gold)	-	[53]
		μg/cigarette	187 ± 22	-	852 ± 67	-	[54]
	Independent	µg/stick	179.4 ± 10.5	183.5 ± 10.1	930 ± 85–1540 ± 153 (literature data)	80.5–88.2	[4]
		µg/cig.	128.50 ± 9.96 (ISO), 210.00 ± 21.71 (HCI)	-	567.00 (ISO), 1534.00 (HCI)	77.34 (ISO), 86.31 (HCI)	[18]
		μg/cigarette	301.46 ± 15.8	-	1059 ± 9.03	-	[21]
		μg/product	156.7 ± 13.8	-	550.5 ± 51.0	-	[23]
		μg/m^3^	3.6 ± 0.4	-	28.9 ± 2.6	-	[33]
		µg/stick	23.034 ± 5.121	-	Comparison with other products	-	[43]
		ng/puff	26,687.7 ± 657.8	-	166,345.0 ± 59,540.1	-	[45]
		mg/m^3^	691 ± 36.5	-	4570 ± 1010	-	[46]
Acrolein	PMI	μg/stick	11.30 ± 2.36/9.44 ± 0.87 (regular)	9.15 ± 0.43/10.91 ± 2.98 (menthol)	154 ± 20/193 ± 21	-	[3]
		μg/unit	10.7 ± 1.7 (in synthetic air)	8.4 ± 1.3 (in nitrogen)	162 ± 3	-	[15]
		μg/article	9.63 ± 0.703	-	156 ± 25.4	93.8	[31]
		μg/stick	9.2 ± 0.865 (regular)	9.36 ± 0.946 (menthol)	177 ± 15.5	94.8–94.71	[32]
		μg/item	8.66 ± 0.483 (regular)	8.3 ± 0.736 (menthol)	155 ± 9.61	94.4–94.6	[49]
		μg/stick	11.3 ± 0.7	-	154 ± 6	93	[52]
		μg/mL	<LOD	-	2.24 (Marlboro Gold)		[53]
		μg/cigarette	18.4 ± 3.1	-	74.7 ± 7.3	-	[54]
	Independent	µg/stick	9.9 ± 1.2	8.9 ± 1.0	89.2 ± 7.3–154.1 ± 13.6 (literature data)	89.5–93.9	[4]
		μg/stick	18.29 ± 3.24	-	185.90 ± 20.59		[9]
		µg/stick	4.01 ± 0.15 (ISO), 6.37 ± 0.32 (HCI)	-	56.7 (ISO), 155.00 (HCI)	92.94 (ISO), 95.89 (HCI)	[18]
		μg/product	4.5 ± 2.62	-	39 ± 3.07	-	[23]
		µg/stick	0.799 ± 0.856	-	Comparison with other products	-	[43]
Formaldehyde	PMI	μg/stick	5.53 ± 0.69/5.22 ± 0.24 (regular)	4.55 ± 0.25/6.19 ± 2.00 (menthol)	56.5 ± 12.1/68.7 ± 7.8	-	[3]
		μg/unit	9.1 ± 1.4 (in synthetic air)	6.1 ± 1.2 (in nitrogen)	87 ± 3		[15]
		μg/article	7.98 ± 0.504	-	85.2 ± 16.7	90.6	[31]
		μg/stick	7.1 ± 0.607 (regular)	7.68 ± 1.234 (menthol)	70.2 ± 6.17	89.89–89.06	[32]
		μg/item	8.97 ± 0.5 (regular)	8.82 ± 1.53 (menthol)	98.9 ± 3.65	90.9–91.1	[49]
		μg/stick	5.53 ± 0.22	-	56.5 ± 3.8	90	[52]
		μg/mL	1.31	-	6.60 (Marlboro Gold)	-	[53]
		μg/cigarette	40.6 ± 5.5	-	17 ± 3.9	-	[54]
	Independent	µg/stick	5.3 ± 0.4	4.7 ± 0.3	29.3 ± 3.8–130.3 ± 10.8 (literature data)	82.9–96.2	[4]
		µg/stick	2.55 ± 0.57	-	16.28 ± 1.63	-	[9]
		μg/cigarette	0.85 ± 0.28	-	3.17 ± 0.33	-	[21]
		μg/product	5.4 ± 1.70	-	29.3 ± 9.27	-	[23]
		μg/m^3^	14.1 ± 0.4	-	27.5 ± 2.2	-	[33]
		µg/stick	1.114 ± 0.810	-	Comparison with other products	-	[43]
		ng/puff	156.9 ± 9.4	-	255.5 ± 60.8	-	[45]
		mg/m^3^	37.8 ± 10.9	-	167 ± 52.1	-	[46]
Glyoxal	Independent	μg/product	15.5 ± 2.50	-	56.2 ± 5.71	-	[23]
		ng/puff	40.7 ± 9.2	-	308.2 ± 92.0	-	[45]
Methylglyoxal	Independent	μg/product	11.3 ± 5.4	-	57.2 ± 9.98	-	[23]
		ng/puff	490.1 ± 69.8	-	982.0 ± 249.0	-	[45]
**Toxic Gases**							
Carbon monoxide (CO)	PMI	mg/stick	0.531 ± 0.068/0.598 ± 0.072 (regular)	0.594 ± 0.110/0.620 ± 0 (menthol)	32.8 ± 2.4/30.7 ± 3.0	-	[3]
		mg/unit	0.54 ± 0.16 (in synthetic air)	<0.530 but ≥0.159 (in nitrogen)	33.4 ± 0.54	-	[15]
		mg/cig	0.25 ± 0.06	-	11.2	99.72	[18]
		mg/article	0.436 ± 0.0811	-	30.2 ± 2.76	98.6	[31]
		μg/stick	<0.067 (regular)	<0.067 (menthol)	30.6 ± 1.83	>99.78	[32]
		mg/item	0.239 (regular)	0.224 (menthol)	27.6 ± 1.3	99.1–99.2	[49]
		mg/stick	0.531 ± 0.021	-	32.8 ± 0.7	98	[52]
		mg/cigarette	0.66 ± 0.03	-	11.1 ± 1.1	-	[54]
	Independent	mg/cig.	0.25 ± 0.06 (ISO), 0.52 ± 0.04 (HCI)	-	11.20 (ISO), 32.70 (HCI)	97.77 (ISO), 98.41 (HCI)	[18]
		mg/product	<LOQ	-	31.5 ± 0.8	-	[23]
		ppm	0.01 ± 0.02	-	0.95 ± 0.41	-	[33]
		mg/cig	0.44 ± 0.04 (regular)	0.43 ± 0.04 (menthol)	33.0 ± 1.8	-	[41]
		mg/m^3^	1090 ± 58.5	-	88,300 ± 16,600		[46]
**Volatile Organic Compounds (VOCs)**							
1,3-butadiene	PMI	μg/stick	0.294 ± 0.042/0.319 ± 0.073(regular)	0.265 ± 0.024/0.411 ± 0.093 (menthol)	63.8 ± 3.5/91.8 ± 11.0	-	[3]
		μg/unit	0.3 ± 0.03 (in nitrogen)	0.3 ± 0.02 (in synthetic air)	98.2 ± 8.4	-	[15]
		μg/article	0.342 ± 0.0347	-	98.5 ± 9.8	99.7	[31]
		μg/article	0.23 ± 0.009 (regular)	0.273 ± 0.028 (menthol)	93 ± 5.55	99.75–99.71	[32]
		µg/item	0.226 ± 0.0703 (regular)	0.209 ± 0.0523 (menthol)	63.8 ± 3.5 (literature data)	-	[37]
		μg/item	0.16 ± 0.0392 (regular)	0.157 ± 0.0178 (menthol)	124 ± 4.73	99.9	[49]
		μg/unit	0.294 ± 0.013	-	63.8 ± 1.1	99.9	[52]
		μg/cigarette	3.07 ± 0.10	-	50.7 ± 2.9	-	[54]
	Independent	µg/stick	0.22 ± 0.02	0.20 ± 0.02	77.0 ± 4.8–116.7 ± 14.3 (literature data)	99.7–99.8	[4]
		μg/stick	NQ (ISO), 0.45 ± 0.03 (HCI)	-	38.5 (ISO), 76.50 (HCI)	99.41 (HCI)	[18]
Acrylonitrile	PMI	μg/stick	0.258 ± 0.041/0.186 ± 0.028 (regular)	0.220 ± 0.014/0.196 ± 0.016 (menthol)	31.9 ± 1.8/31.6 ± 2.3	-	[3]
		μg/unit	0.2 ± 0.02 (in nitrogen)	0.2 ± 0.02 (in synthetic air)	26.1 ± 4.3	-	[15]
		μg/article	0.158 ± 0.0122	-	24.5 ± 3.52	99.4	[31]
		μg/article	<0.107 (regular)	0.112 ± 0.039 (menthol)	22.5 ± 1.73	>99.52–99.5	[32]
		µg/item	0.133 ± 0.0348 (regular)	0.120 ± 0.02801 (menthol)	31.9 ± 1.8 (literature data)	-	[37]
		μg/item	0.134 (regular)	0.135 (menthol)	22.4 ± 0.808	99.4	[49]
		μg/stick	0.258 ± 0.013	-	31.9 ± 0.6	99	[52]
	Independent	μg/stick	ND (ISO)/ 0.21 ± 0.01 (HCI)	-	26.4 (ISO)/67 (HCI)	-	[18]
Benzene	PMI	μg/stick	0.649 ± 0.074/0.575 ± 0.072 (regular)	0.640 ± 0.040/0.628 ± 0.073 (menthol)	97.6 ± 4.7/100.4 ± 2.8	-	[3]
		μg/unit	0.5 ± 0.07 (in nitrogen)	0.6 ± 0.06 (in synthetic air)	90.7 ± 12.5	-	[15]
		μg/article	0.544 ± 0.0312	-	81.1 ± 8.78	99.3	[31]
		μg/article	0.483 ± 0.023 (regular)	0.561 ± 0.072 (menthol)	83.1 ± 3.02	99.42–99.32	[32]
		µg/item	0.529 ± 0.151 (regular)	0.487 ± 0.0869 (menthol)	97.6 ± 4.7 (literature data)	-	[37]
		μg/item	0.372 ± 0.0737 (regular)	0.41 ± 0.04 (menthol)	74 ± 2.74	99.5–99.4	[50]
		μg/stick	0.649 ± 0.023	-	97.6 ± 1.5	99	[52]
		μg/cigarette	1.1 ± 0.1	-	50.6 ± 0.5	-	[54]
	Independent	µg/stick	0.63 ± 0.07	0.54 ± 0.05	49.7 ± 7.7–98.3 ± 4.3 (literature data)	98.8–99.4	[4]
		μg/stick	0.12 ± 0.01 (ISO), 0.61 ± 0.04 (HCI)	-	45.7 (ISO), 104.00 (HCI)	99.74 (ISO), 99.41 (HCI)	[18]
		μg/stick	0.534 ± 0.040	-	Comparison with other products	-	[43]
Toluene	PMI	μg/stick	2.59 ± 0.43/1.61 ± 0.17 (regular)	2.39 ± 0.16/1.67 ± 0.37 (menthol)	188 ± 11/198.8 ± 10.9	-	[3]
		μg/unit	1.9 ± 0.3 (in nitrogen)	2.0 ± 0.2 (in synthetic air)	158 ± 24	-	[15]
		μg/article	1.82 ± 0.163	-	137 ± 16.9	98.7	[31]
		μg/article	1.4 ± 0.054 (regular)	1.65 ± 0.227 (menthol)	143 ± 6.74	99.02–98.85	[32]
		µg/item	1.52 ± 0.522 (regular)	1.33 ± 0.3527 (menthol)	188 ± 11 (literature data)	-	[37]
		μg/item	1.04 ± 0.223 (regular)	1.17 ± 0.115 (menthol)	97.7 ± 2.35	98.9–98.8	[49]
		μg/stick	2.59 ± 0.14	-	188 ± 4	99	[52]
		μg/cigarette	4.15 ± 0.41	-	83.1 ± 2.9	-	[54]
	Independent	µg/stick	2.15 ± 0.37	1.96 ± 0.23	86.2 ± 11.0–176.2 ± 15.7 (literature data)	97.6–98.8	[4]
		μg/stick	0.84 ± 0.05 (ISO), 2.48 ± 0.18 (HCI)	-	73.6 (ISO), 208.00 (HCI)	98.86 (ISO), 98.81 (HCI)	[18]
		μg/stick	1.203 ± 0.332	-	Comparison with other products	-	[43]
		μg/cigarette	1.22 ± 0.05	-	127 ± 8.44	+	[55]
**Tobacco-specific nitrosamines (TSNAs)**							
NNK	PMI	ng/stick	6.7 ± 0.6/10.1 ± 0.4 (regular)	5.9 ± 0.4/7.9 ± 1.1 (menthol)	266 ± 15/257 ± 39	-	[3]
		ng/article	10.3	-	95.18	-	[8]
		ng/article	6.75± 0.493	-	264 ±26.4	97.4	[31]
		ng/article	9 ± 0.485 (regular)	6.92 ± 0.902 (menthol)	232 ± 7.31	96.12–97.02	[32]
		ng/stick	9.47 ± 0.479 (regular)	7.94 ± 0.293 (menthol)	210 ± 9.17	95.5–96.2	[49]
		ng/stick	6.67 ± 0.19	-	266 ± 5	97	[51]
		ng/cigarette	15 ± 3	-	103 ± 8	-	[54]
	Independent	ng/stick	5.65 ± 0.30	-	188.27 ± 19.81	-	[9]
		μg/stick	3.50 ± 0.17 (ISO), 7.30 ± 0.34 (HCI)	-	85.50 (ISO), 243 (HCI)	95.91 (ISO), 97.00 (HCI)	[18]
		ng/product	2.5 ± 0.34	-	201.0 ± 17.10	-	[23]
		ng/cig	12.3 ± 1.5 (regular)	13.8 ± 2.6 (menthol)	250.4 ± 13.7	-	[41]
		μg/stick	4.538 ± 0.893	-	Comparison with other products	-	[43]
NNN	PMI	ng/stick	17.2 ± 1.25/10.3 ± 0.4 (regular)	13.7 ± 1.21/7.7 ± 1.0 (menthol)	309 ± 41/268 ± 50	-	[3]
		ng/article	18.8	-	162.9	-	[8]
		ng/article	10.2 ± 0.486	-	283 ± 27.8	96.4	[31]
		ng/article	15.2 ± 1.55 (regular)	9.5 ± 1.62 (menthol)	277 ± 39.7	94.51–96.57	[32]
		ng/stick	12.4 ± 0.381 (regular)	8.28 ± 0.22 (menthol)	247 ± 5.03	95.0–96.7	[49]
		ng/stick	17.2 ± 0.4	-	309 ± 13	94	[52]
		ng/cigarette	24 ± 2	-	134 ± 8	-	[54]
	Independent	ng/stick	6.52 ± 0.51	-	240.01 ± 19.01	-	[9]
		ng/cigarette	5.00 ± 0.32 (ISO), 10.50 ± 0.46 (HCI)	-	92.1 (ISO), 276.00 (HCI)	94.57 (ISO), 96.20 (HCI)	[18]
		ng/prod	3.2 ± 0.64	-	240.8 ± 13.20	-	[23]
		ng/cig	19.2 ± 2.1 (regular)	24.9 ± 3.5 (menthol)	311.1 ± 24.3	-	[41]
		μg/stick	12.650 ± 1.933	-	Comparison with other products	-	[43]
**Polyaromatic hydrocarbons (PAHs)**							
Benzo[α]pyrene	PMI	ng/stick	<1.00/1.19 ± 0.0 (regular)	1.29 ± 0.10/1.08 ± 0.09 (menthol)	14.2 ± 0.3/13.7 ± 0.8	-	[3]
		ng/unit	0.60 ± 0.09 (in nitrogen)	0.61 ± 0.11 (in synthetic air)	17.3 ± 0.9	-	[15]
		ng/article	0.939 ± 0.0796	-	15 ± 1.3	93.7	[31]
		ng/article	1.1 ± 0.17 (regular)	0.7 ± 0.07 (menthol)	16 ± 0.9	93.13–95.63	[32]
		ng/item	0.556 ± 0.0369 (regular)	0.657 ± 0.0303 (menthol)	14.5 ± 0.2	96.2–95.5	[49]
		ng/stick	<1.00	-	14.2 ± 0.1	93	[52]
		ng/cigarette)	<0.27	-	6.94 ± 1	-	[54]
	Independent	ng/cig.	NQ (ISO, HCI)	-	6.73 (ISO), 16.20 (HCI)	-	[18]
		pg/puff	25.6 ± 13.8	-	457.6 ± 114.5	-	[45]
Naphthalene	PMI	ng/article	7.34 ± 1.18 (regular)	5.94 ± 0.9 (menthol)	1197 ± 83.1	99.39–99.5	[32]
		µg/item	0.0116 ± 0.00357 (regular)	0.00885 ± 0.00339 (menthol)	-	-	[37]
		ng/cigarette)	5.58 ± 0.54	-	822 ± 130	-	[54]
**Phenolics**							
Phenol	PMI	μg/stick	1.16 ± 0.12/1.51 ± 0.23 (regular)	1.60 ± 0.4/1.00 ± 0.17 (menthol)	13.6 ± 0.9/13.2 ± 0.9	-	[3]
		μg/article	1.12 ± 0.0849	-	14 ± 1.86	92	[31]
		μg/article	0.941 ± 0.134 (regular)	0.812 ± 0.088 (menthol)	14.4 ± 0.777	93.47–94.36	[32]
		µg/item	1.78 ± 0.894 (regular)	0.770 ± 0.2990 (menthol)	13.6 ± 0.9 (literature data)	-	[37]
		μg/stick	0.794 ± 0.0832 (regular)	0.905 ± 0.0851 (menthol)	14 ± 0	94.3–93.5	[49]
		μg/stick	1.16 ± 0.04	-	13.6 ± 0.3	91	[52]
		μg/cigarette	0.49 ± 0.06	-	11.8 ± 0.5	-	[54]
	Independent	μg/cig.	NQ (ISO), 1.20 ± 0.05 (HCI)	-	7.04 (ISO), 14.80 (HCI)	95.8 (HCI)	[18]
Catechol	PMI	μg/stick	16.3 ± 1.5/16.4 ± 0.6 (regular)	17.1 ± 1.1/12.8 ± 1.3 (menthol)	91.4 ± 5.6/88.7 ± 2.6	-	[3]
		μg/unit	14.7 ± 1.1 (in nitrogen)	14.3 ± 0.5 (in synthetic air)	84.2 ± 1.2	-	[15]
		μg/article	14.4 ± 0.68	-	89.8 ± 7.14	84	[31]
		μg/article	12.9 ± 0.941 (regular)	12.7 ± 0.949 (menthol)	98.1 ± 7.34	86.85–87.05	[32]
		µg/item	14.7 ± 3.94 (regular)	12.9 ± 3.059 (menthol)	91.4 ± 5.6 (literature data)	-	[37]
		μg/item	11.1 ± 0.433 (regular)	11.4 ± 0.608 (menthol)	93.4 ± 5.85	88.1–87.8	[49]
		μg/stick	16.3 ± 0.5	-	91.4 ± 1.8	82	[52]
		μg/cigarette	6.25 ± 0.48	-	40.6 ± 1.0	-	[54]
**Toxic Metals**							
Cadmium	PMI	ng/stick	<0.350/<0.350 (regular)	<0.350/<0.350 (menthol)	161 ± 4/122 ± 12	-	[3]
		ng/article	<0.28	-	92.9 ± 10.04	>99.7	[31]
		ng/article	<0.09 (regular)	<0.28 (menthol)	99.4 ± 4.84	>99.91–>99.72	[32]
		ng/item	<0.075 (regular)	<0.075 (menthol)	102 ± 7.01	99.9	[49]
		ng/stick	<0.350	-	161 ± 1	99	[52]
		ng/cigarette	0.61 ± 0.03	-	31.5 ± 13.1	-	[54]
Lead	PMI	ng/stick	<3.35/<3.35 (regular)	<3.35/<3.35 (menthol)	37.0 ± 0.7	-	[3]
		ng/article	<1.62	-	32.1 ± 4	>95	[31]
		ng/article	<1.62 (regular)	<0.49 (menthol)	<25.7	-	[32]
		ng/stick	<0.5 (regular)	<0.5 (menthol)	29.8 ± 1.21	98.3	[49]
		ng/stick	<3.35	-	37 ± 0.2	91	[52]
		ng/cigarette	<0.7	-	32.0 ± 2.0	-	[54]
Nickel	PMI	ng/stick	<0.55/<0.55 (regular)	<0.55/0.88 (menthol)	<0.55/1.30	-	[3]
		ng/article	<15.9	-	<43.1	-	[32]
		ng/item	<5	<5	<3	-	[49]
		ng/stick	<0.550	-	<0.550	-	[52]
		ng/cigarette	<0.7	-	<2	-	[54]
Mercury	PMI	ng/stick	1.17 ± 0.05/1.02 ± 0.05 (regular)	1.34 ± 0.18/1.12 ± 0.19 (menthol)	4.80 ± 0.13/4.17 ± 0.74	-	[3]
		ng/article	2.04 ± 0.104	-	4.77 ± 0.669	57.1	[31]
		ng/article	2.11 ± 0.071 (regular)	1.88 ± 0.19 (menthol)	4.36 ± 0.36	51.61–56.88	[32]
		ng/item	1.54 ± 0.247 (regular)	1.81 ± 0.382 (menthol)	5.66 ± 0.332	72.7–68.0	[49]
		ng/stick	1.17 ± 0.02	-	4.8 ± 0.04	76	[52]

## 5. Toxicological and Public Health Implications

The previous sections summarized the occurrence and analytical determination of the principal classes of HPHCs identified in IQOS aerosols. The following sections focus on the toxicological interpretation of these findings, by integrating the available evidence according to the major biological outcomes associated with the detected compounds, including carcinogenic, respiratory, cardiovascular, and systemic effects.

Toxicological relevance of IQOS remains a controversial topic, which cannot be addressed based only on comparison of percentage decreases in toxicant emissions in relation to conventional cigarette smoke. Even though aerosols from heated tobacco products contain lower levels of most combustion-derived toxicants, health risk depends on various factors such as toxicity of a compound, delivery dose, repetitive inhalation, inhalation bioavailability, interaction effects, and user behavior [56]. Hence, the previously mentioned HPHCs continue to be important for risk assessment, regardless of their decreased level in comparison with conventional cigarette smoke [23].

### 5.1. Carcinogenic Effect

Carcinogenic potential remains one of the key aspects for evaluation of tobacco-related emissions. Even though IQOS aerosols contain generally lower concentrations of many combustion-derived toxicants, some chemicals with well-established carcinogenic effect still remain detectable [5,38,56], including tobacco-specific nitrosamines (TSNAs), especially NNK and NNN, which have a high affinity for DNA adduct formation and cause mutagenesis after activation [41]. Contrary to many other combustion-derived toxicants, TSNAs are mainly transferred during aerosol formation from tobacco itself rather than byproducts of the process.

Furthermore, certain volatile organic compounds (VOCs) including benzene and 1,3-butadiene, and polycyclic aromatic hydrocarbons (PAHs)—particularly benzo[a]pyrene—remain relevant compounds for assessment of carcinogenic risk even though their concentrations in IQOS aerosols are notably lower compared with conventional cigarette smoke—a trend also observed for other heat-not-burn devices [57,58,59,60,61,62,63,64]. Additionally, some carbonyls, which include acrolein, formaldehyde, and acetaldehyde, are well known for their carcinogenic potential [21]. Because they can form as result of thermal decomposition of nicotine, glycerol, and other tobacco constituents, their concentrations may vary substantially depending on puffing conditions.

Based on currently available information, aerosols from IQOS devices expose consumers to lower concentrations of carcinogens than in conventional cigarette smoke, but they do not completely avoid these chemicals. However, evaluation of toxicological significance of this phenomenon remains complicated without access to long-term epidemiological data on heated tobacco use.

### 5.2. Respiratory Toxicity

Because aerosols of heat-not-burn devices enter the body through respiration, evaluation of respiratory toxicity becomes an important part of risk assessment of heated tobacco products. Even though heated tobacco aerosols differ from conventional cigarette smoke in their composition and particulate matter, they still contain reactive compounds which can cause oxidative stress, inflammation, and epithelial damages [56,58,64,65].

Particularly interesting are carbonyls, as they are highly reactive compounds and can induce oxidative stress, compromise epithelial barrier integrity, and induce inflammatory response [30]. Even though concentrations of these chemicals may be lower than those detected in conventional cigarette smoke, their presence is particularly relevant, as repetitive exposure might lead to development of biological effects.

Physical and chemical properties of IQOS aerosols can also influence their distribution pattern in the respiratory system. Contrary to combustion-derived smoke particles, IQOS aerosols consist mainly of semi-volatiles consisting of water, nicotine, glycerol, and other dissolved organic compounds and can penetrate into the respiratory tract [66,67]. Several studies, in which aerosols from heated tobacco were compared with conventional cigarette smoke, found that cytotoxic and inflammatory response to heated aerosols was attenuated in comparison with smoke from conventional cigarettes [45,52,65,68]. However, interpretation of this information becomes difficult due to significant methodological variability in these investigations.

Overall, currently available evidence suggests that IQOS aerosols may have a lower toxicological potential than conventional cigarette smoke for several endpoints; however, the magnitude and long-term relevance of these differences remain uncertain.

### 5.3. Cardiovascular Effects

Cardiovascular effects remain a major aspect in evaluation of HTP because conventional cigarettes are known to be associated with oxidative stress, endothelial dysfunction, increased thrombosis, and other effects which may induce heart disease. Many toxicants contained in IQOS aerosols may induce similar effects, even though their concentrations remain lower than in conventional cigarette smoke [56].

First and foremost, carbon monoxide remains a toxicant of particular importance in this respect since its concentration in IQOS aerosols is dramatically reduced because of the lack of sustained burning in these devices [14,32]; this becomes a key difference between heated tobacco products and combustible cigarettes.

However, cardiovascular effects cannot be analyzed based on reduction in CO emissions exclusively. For example, certain reactive aldehydes including acrolein can induce oxidative stress, endothelial dysfunction, and promote inflammation via specific signaling pathways [20,30]. Trace metals including cadmium, lead, nickel, and mercury may also induce cardiovascular and systemic effects if accumulated due to prolonged exposure [48,58].

Finally, although overall content of reactive oxygen species and free radicals is significantly lower than in conventional cigarette smoke, their presence still remains relevant for evaluation of cardiovascular toxicity [10,11].

Therefore, available information on IQOS aerosols indicates their reduced cardiovascular toxicity when compared with conventional cigarette smoke; however, long-term implications of exposure remain unclear.

### 5.4. Neurotoxicity and Systemic Effects

In addition to cardiovascular and respiratory effects, several toxicants found in IQOS aerosols might induce systemic and neurobehavioral effects upon prolonged inhalation. There are certain volatile organic compounds and trace metals among these compounds that might be distributed beyond the respiratory system and that affect multiple biological systems [24,43,44].

For instance, among VOCs there are such toxicants as toluene, styrene (known to induce neurobehavioral and irritative effects), and acrylonitrile, which may cause systemic toxicity because of reactive metabolites produced during its metabolism. Despite that their concentrations are much lower than in conventional cigarette smoke, their continued presence remains relevant for cumulative exposure analysis.

Trace metals are studied because they have a tendency to accumulate in different biological tissues and induce toxic effects. Among them cadmium remains associated with nephrological and cardiovascular effects, while lead causes neurobehavioral toxicity [49,51]. Toxicological significance of these effects depends on several factors: concentration, bioavailability, and exposure duration [62,63].

Finally, it needs to be emphasized that although nicotine is not the main focus of this study, its role should also not be underestimated. IQOS devices are efficient at delivering nicotine; sometimes, their nicotine delivery rate approaches that of conventional cigarettes in cases of intensive usage [69]—having an important impact on addiction.

Notably, users are exposed to complex mixtures of toxicants, which might interact with each other and produce additive or even synergistic effects.

### 5.5. Implications for Exposure and Risk Assessment

Evaluating HTP presents several challenges due to difficulties associated with the distinction between emission reduction and health risk reduction. Although laboratory analysis of aerosols can provide necessary data for evaluating aerosol composition and yields, it is unable to provide information about long-term health effects [16].

Most studies reviewed in the current article consistently showed lower toxicant levels in IQOS aerosols compared with conventional cigarette smoke, particularly in relation to CO, PAHs, and some volatile organic compounds [3,31]. Lower toxicant levels do not necessarily correlate proportionally with decreased health risk, as patterns of exposure, toxicant interactions, and individual susceptibility may vary greatly between users.

Moreover, biological activity of a toxicant can be evaluated with the help of biomarkers of exposure, which reflects exposure to certain substances that are actively being metabolized. Several studies investigating biomarkers of exposure in smokers who switched from conventional cigarettes to HTPs found reduction in biomarkers related to CO, VOCs, TSNAs, and acrolein [30,70]; however, interpretation of these results may be complicated due to dual use of different tobacco products and other factors.

Another issue here is the absence of long-term epidemiological evidence regarding HTPs. Current data is mostly represented by analyses of emissions, in vitro systems, short-term exposure studies, and biomarkers; however, it takes a much longer period of time for the development of diseases like cancer, chronic obstructive pulmonary disease, and cardiovascular disease [25,71].

Significant heterogeneity in methodology used in aerosol evaluations also hampers analysis of results. Various puffing regimens, aerosol sampling techniques, device generations, consumables, and analytical methodologies used to analyze aerosol composition may influence the results obtained. Finally, the majority of available information refers to earlier IQOS models, like THS 2.2, while newer models are still poorly studied.

Based on the above information, it can be stated that aerosols from IQOS products contain lower concentrations of combustion-derived toxicants compared with conventional cigarette smoke. Nevertheless, these aerosols remain mixtures of biologically active chemicals, and more evidence, especially long-term epidemiological evidence, will be needed to estimate their health implications in greater detail.

### 5.6. Public Health and Regulatory Implications

The introduction of HTPs created many new challenges associated with the regulation and monitoring of these products and their effects on public health. Products like IQOS are positioned by manufacturers as alternatives to conventional cigarette smoke because they create aerosols through heating rather than combustion. This leads to lower emissions of traditional toxicants from conventional cigarette smoke [12,58]. However, their public health implications are far from clear.

One of the key issues for evaluation of heated tobacco products is related to heterogeneity of usage patterns among consumers. People may use such products exclusively, or together with regular cigarettes, depending on many factors, including smoking history, age, product availability, nicotine dependence, and consumer preferences [72]. Such diversity makes assessment of exposure patterns quite complicated.

It becomes critical to distinguish reduced emissions and reduced health risks associated with HTPs. Even though numerous studies confirm lower concentrations of many combustion-derived toxicants in IQOS aerosols compared with conventional cigarette smoke, users are still exposed to certain chemicals like nicotine, carbonyls, VOCs, TSNAs, phenolic compounds, and trace metals [3,9,31]. Thus, aerosol chemistry should not be regarded as a key determinant for health risks alone.

Regulatory authorities emphasize the importance of this aspect as well. For example, WHO repeatedly stated that heated tobacco products emit toxicants and must remain regulated under tobacco control measures [1,70]. The FDA authorized modified exposure claims for certain models of IQOS devices, but strictly distinguished reduced exposure from health benefit in its guidelines [25,26].

Another important aspect to consider is the dynamic nature of technology. Rapid development of device design, aerosol delivery methods, and consumable compositions requires continuous characterization of aerosols and toxicants emitted by heated tobacco products.

From a public health standpoint, ongoing surveillance will be vital for evaluation of all heated tobacco products in the future. Not only laboratory studies focused on aerosol emissions, but also toxicological studies, biomarker investigation, behavioral data, and long-term epidemiological studies will be necessary to evaluate their impact on public health [72,73].

Thus, the currently available information suggests that IQOS products emit aerosols which differ from conventional cigarette smoke, and that they generally emit fewer toxicants. However, because these aerosols remain complex mixtures, continuous independent research is needed to evaluate their health risks in greater detail.

The present review has several limitations that should be considered when interpreting its findings. The available evidence remains heterogeneous, reflecting differences in device generations, puffing regimens, aerosol collection strategies, analytical methodologies, and reporting units across studies. In addition, much of the current literature focuses on earlier IQOS generations, while newer products remain comparatively underexplored. The review process itself also has limitations, including restriction to studies indexed in the Web of Science Core Collection database and the absence of a formal risk-of-bias assessment, which was not performed as the included publications are analytical chemistry studies, not clinical or epidemiological investigations, for which validated assessment tools have been developed. Nevertheless, methodological quality was considered qualitatively during evidence synthesis by evaluating the transparency of aerosol generation protocols, analytical methodologies, reporting completeness, institutional affiliation, funding sources, and declared conflicts of interest. Consequently, the findings presented in this review should be interpreted as a synthesis of the currently available analytical evidence rather than as a definitive assessment of all heated tobacco products or all possible exposure scenarios.

## 6. Conclusions

As shown by the current review, IQOS aerosols are chemically heterogeneous and their evaluation requires going beyond the conventional toxicant panels designed to assess conventional cigarette smoke toxicity. While maintaining the relevance of WHO 9 priority toxicants for regulatory purposes, the available evidence indicates that IQOS aerosols have a wider chemical composition, which requires comprehensive chemical characterization and further toxicological investigation.

Across the studies included in this review, IQOS aerosols consistently exhibited lower measured concentrations of numerous toxicants, especially carbon monoxide, PAHs, and various volatile organic compounds. On the other hand, carbonyls, tobacco-specific nitrosamines, phenolics, and trace metals have been detected consistently in several studies and hence remain relevant targets for toxicological assessment and analytical monitoring.

The substantial methodological heterogeneity among published studies (including differences in IQOS generation, puffing regimens, aerosol collection strategies, analytical methodologies, and reporting units) limits direct quantitative comparisons across studies. However, based on the available data, this review proposes an expanded panel of twenty priority toxicants that may facilitate a more comprehensive chemical characterization of IQOS aerosols and contribute to future harmonization of analytical methodologies used for heated tobacco product assessment.

Overall, IQOS aerosols differ substantially in terms of chemical composition from conventional cigarette smoke and generally contain lower levels of many combustion-related toxicants, but remain chemically complex mixtures containing biologically active compounds. The proposed expanded toxicant panel provides a structured framework for future analytical monitoring and aerosol characterization. Further progress in understanding the health implications of heated tobacco product use will require integration of analytical chemistry with toxicological, clinical, biomarker, and epidemiological evidence, which lies beyond the scope of the present review.

## Figures and Tables

**Table 1:** Definition of PICO strategy applied for the present work.

P (Problem)	Insufficient data regarding the level of toxicants in HTPs and lack of common approach for the evaluation of the HTP emissions
I (Intervention)	Development of an extended list of toxicants, supported by the available literature data
C (Comparison)	Either reference cigarette or commercially available classic cigarettes
O (Outcome)	Improvement of HTPs content monitorization and a more reliable evaluation of their potential health effects

**Table 2:** Expanded panel of twenty priority toxicants proposed for IQOS aerosol monitoring, selected according to four complementary criteria: toxicological relevance, frequency of detection in the reviewed literature, regulatory significance, and analytical feasibility.

Chemical Class	Proposed Constituents
Toxic gases	Carbon monoxide
Carbonyls	Formaldehyde, acetaldehyde, acrolein, glyoxal, methylglyoxal
VOCs	Benzene, 1,3-butadiene, acrylonitrile, toluene
TSNAs	NNK, NNN
PAHs	Benzo[a]pyrene, naphthalene
Phenolics	Phenol, catechol
Metals	Cadmium, lead, nickel, mercury

## Data Availability

No new data were created or analyzed in this study. Data sharing is not applicable to this article.

## Supplementary Materials

The following supporting information can be downloaded at: https://www.mdpi.com/article/10.3390/toxics14070614/s1, Figure S1: PRISMA flow diagram; Table S1: Characteristics of the studies included in the systematic review and used for development of the proposed toxicant panel; Table S2: Additional publications used as supporting evidence but not included in the systematic synthesis.

## Abbreviations

The following abbreviations are used in this manuscript (alphabetical order):

CO	Carbon monoxide
FDA	(U.S.) Food and Drug Administration
GC-MS	Gas chromatography–mass spectrometry
HCI	Health Canada Intense
HCN	Hydrogen cyanide
HPHCs	Harmful and potentially harmful constituents
HPLC	High-performance liquid chromatography
HTPs	Heated tobacco products
ICP-MS	Inductively coupled plasma mass spectrometry
ISO	International Organization for Standardization
LC-MS/MS	Liquid chromatography-tandem mass spectrometry
LOD	Limit of detection
LOQ	Limit of quantification
ND	Not detected
NNK	(4-(methylnitrosamino)-1-(3-pyridyl)-1-butanone)
NNN	(N′-nitrosonornicotine)
PAHs	Polycyclic aromatic hydrocarbons
PICO	Problem, Intervention, Comparison, Outcome
PRISMA	Preferred Reporting Items for Systematic Reviews and Meta-Analyses
THS	Tobacco heating system
TSNAs	Tobacco-specific nitrosamines
VOCs	Volatile organic compounds
WHO	World Health Organization
