# Supplementary material for: Beyond the WHO Priority Toxicants: A Systematic Review of Harmful and Potentially Harmful Constituents in IQOS Aerosols
Source: Toxics. 2026 Jul 13;14(7):614. doi: 10.3390/toxics14070614 (PMC13417897; doi:10.3390/toxics14070614)
Supplement: Supplementary file 1 [file toxics-14-00614-s001.zip › Supplementary material 2.pdf]

Supplementary material 2

Beyond the WHO Priority Toxicants: A Systematic Review of Harmful and Potentially Harmful Constituents in IQOS Aerosols

Roxana Ioana Matei [1], Anda Maria Baroi [1], Toma Fistos [1], Irina Fierascu [1,2,†] and Radu Claudiu Fierascu [1,3,4,*,†]

[1] National Institute for Research & Development in Chemistry and Petrochemistry—ICECHIM Bucharest,
202 Splaiul Independenței, 060021 Bucharest, Romania; roxana.brazdis@icechim.ro (R.I.M.); anda.baroi@icechim.ro (A.M.B.); toma.fistos@icechim.ro (T.F.); irina.fierascu@icechim.ro (I.F.)
[2] Faculty of Horticulture, University of Agronomic Sciences and Veterinary Medicine of Bucharest, 59 Marasti Blvd, District 1, 011464 Bucharest, Romania
[3] Faculty of Chemical Engineering and Biotechnologies, National University of Science and Technology Politehnica Bucharest, 1-7 Gheorghe Polizu St., 011061 Bucharest, Romania
[4] Academy of Romanian Scientists, 3 Ilfov Str., 050044 Bucharest, Romania
* Correspondence: fierascu.radu@icechim.ro
† These authors contributed equally to this work.
[#] Authors with equal contribution to the present work

This Supplementary Material provides additional information supporting the systematic review process and the development of the proposed expanded toxicant panel for IQOS aerosols. To improve the transparency of the evidence synthesis, the publications considered during the review are presented according to their role in the study.

Supplementary Table S1 summarizes the characteristics of the analytical studies, including institutional affiliation, funding source, declared conflicts of interest (where available), investigated analyte classes, and analytical methodologies.

Supplementary Table S2 lists the additional publications consulted to support the interpretation of toxicological relevance, analytical methodologies, and regulatory aspects, but which were not used for toxicant selection.

Table S1. Characteristics of the studies included in the systematic review and used for development of the proposed toxicant panel.

No.	Reference	Institutional affiliation	Funding source	Competing interests (as declared)	IQOS generation	Puffing regimen	Main analyte classes	Analytical techniques
1	Schaller et al. [3]	PMI (IQOS manufacturer)	Not reported	"The work reported in this publication involved a candidate Modified Risk Tobacco Product developed by Philip Morris International (PMI) and was solely funded by PMI. All authors are (or were) employees of PMI R&D or worked for PMI R&D under contractual agreements."	THS 2.2, regular and menthol sticks, different versions	Different regiments, including ISO (volume 55 mL, puff duration of 2 s, puff frequency - one puff every 60 s) and HCI (volume 55 mL, puff duration of 2 s, puff frequency - one puff every 30 s).	1. Carbonyls<br>2. Toxic gases (CO)<br>3. VOCs<br>4. TSNAs<br>5. PAHs<br>6. Phenolics<br>7. Toxic metals	1, 4. LC-MS/MS<br>2. carbon monoxide (CO) meter<br>3, 5, 6. GC/MS<br>7. ICP-MS
2.	Mallock et al. [4]	Independent	National research funds	"The authors declare that they have no conflict of interest."	THS2.2	HCI: volume 55 mL, puff duration of 2 s, puff frequency - one puff every 30 s.	1. Carbonyls<br>2. VOCs	1. LC, LC-MS/MS<br>2. GC-MS
3.	Jaccard et al. [8]	PMI (IQOS manufacturer)	Funded by PMI	The authors have "nothing to disclose" according the transparecy documents	THS2.2	HCI	1. TSNAs	1. LC-MS/MS
4.	Wang et al. [9]	Independent	National research funds	"The authors declare that they have no known competing financial interests or personal relationships that could have appeared to influence the work reported in this paper."	THS 2.2, menthol sticks	HCI: puff volume of 55 mL, a puff duration of 2 s, a puff frequency of one puff every 30 s, and complete (100%) blocking of ventilation holes.	1. Carbonyls<br>2. TSNAs<br>3. Untargeted analysis	1, 2. UPLC-MS/MS (liquid chromatography/ ion mobility-quadrupole time-of-flight mass spectrometry)<br>3. UPLC-QTOF-MS (ultra-high performance liquid chromatography/ion mobility-

5.	Cozzani et al. [15]	Collaboration between independent researchers and PMI-affiliated	Funded by PMI	"T. McGrath, B. Mahler, M. Nordlund, M. Smith, J.P. Schaller, and G. Zuber are Philip Morris International employees (PMI)."	THS 2.2	HCI: 55 mL puff volume, 2 s puff duration, 30 s puff frequency.	1. Carbonyls<br>2. Toxic gases (CO)<br>3. VOCs<br>4. PAHs<br>5. Phenolics	quadrupole time-of-flight mass spectrometry)<br>All analyses were performed using validated Labstat test methods<br>1, 3, 4, 5 – GC-TCD (gas chromatography using a thermal conductivity detector).<br>2. CO/CO_2 Analyzer for FTIR
6.	Li et al. [18]	Independent	National research funds	"The authors declare there is no conflicts of interest regarding the publication of this paper."	THS 2.2	ISO – 35 mL puff volume every 60s, puff duration of 2s, no blocking of filter ventilation; 6 puffs number – 6;<br>HCI – 55 mL puff volume every 30s, puff duration 2s, 100% blocking of filter ventilation; puffs number – 12.	1. Carbonyls<br>2. Toxic gases (CO)<br>3. VOCs<br>4. TSNAs<br>5. Phenolics	ISO methods:<br>1, 4, 5. HPLC<br>2. nondispersive infrared photometry<br>3. GC-MS
7.	Salman et al. [21]	Independent	National research funds	"The authors declare the following competing financial interest(s): AS is a paid consultant in litigation against the tobacco industry and is named on a patent application for a device that measures the puffing behavior of electronic cigarette users."	THS 2.2 (IQOS)	HCI – 55 mL puff volume every 30s, puff duration 2s	1. Carbonyls	1. HPLC

8.	Davigo et al. [23]	Independent	National research funds	"None declared."	IQOS 3 DUO	World Health Organization standard operating procedure for intense smoking of cigarettes	1. Carbonyls<br>2. Toxic gases (CO)<br>3. TSNAs	1. Liquid chromatography with diode array detection (LC-DAD)<br>2. nondispersive infrared analyze<br>3. LC-MS
9.	Jaccard et al. [31]	PMI (IQOS manufacturer)	Not reported	The authors have "nothing to disclose" according the transparecy documents	THS 2.2	HCI	1. Carbonyls<br>2. Toxic gases (CO)<br>3. VOCs<br>4. TSNAs<br>5. PAHs<br>6. Phenolics<br>7. Toxic metals	ISO 17025 accredited methods, according Tobacco Reporting Regulations. SOR/200e273. Registration 2000-06-26. Part 3: Emissions from designated tobacco products, except:<br>4. LC-MS<br>5. GC-MS
10.	Maeder & Jeannet [32]	PMI (IQOS manufacturer)	Funder - Philip Morris Products S.A.	"The authors declare the following competing financial interest(s): The work reported in this publication involved products developed by Philip Morris Products S.A., which is the sole source of funding and sponsor of this research. Both authors are employees of Philip Morris Products S.A. research and development. Both authors own stock in Philip Morris	THS 2.2, regular and menthol sticks	HCI, puff volume, 55 mL; puff duration, 2 s; puff interval 30 s; ventilation blocking 100.	1. Carbonyls<br>2. Toxic gases (CO)<br>3. VOCs<br>4. TSNAs<br>5. PAHs<br>6. Phenolics<br>7. Toxic metals	1. reverse-phase HPLC<br>2. non-dispersive infra-red analyzer<br>3, 5. GC-MS<br>4. LC-MS/MS<br>6. reversed-phase gradient liquid chromatography, using selective fluorescence

				International. SM is a listed inventor on some patents held by Philip Morris Products S.A.”				detection for quantification<br>7. ICP-MS
11.	Meišutovič-Akhtarieva et al. [33]	Independent	Funder - Philip Morris Products SA	Acknowledgement - “This research was funded by an Investigator-Initiated Study award by Philip Morris Products SA, Switzerland (IIS.PMI.2017.16). The study protocol was written by the investigator, who also conducted the study. Philip Morris Products SA had no involvement in the study conduct, data analysis, and writing of the manuscript.”	THS 2.2	The experiment aimed to research the quantitative effects of environmental variables including ventilation intensity (V) as air changes per hour (0.2, 0.5, or 1), THS use intensity (I) as number of parallel users (1, 3, or 5), relative humidity (RH, 30, 50 or 70%), and distance to the bystander (D, 0.5, 1, or 2m) onto pollutant concentration variations in a chamber.	1. Carbonyls<br>2. Toxic gases (CO)	1. HPLC<br>2. electrochemical sensor
12.	Hofer et al. [37]	PMI (IQOS manufacturer)	Funded by PMI	No Conflict of interests/transparency documents provided	THS 2.4	HCI, puff volume, 55 mL; puff duration, 2 s; puff interval 30 s; ventilation blocking 100.	1. VOCs<br>2. PAHs<br>3. Phenolics	1-3. GC-MS
13.	Bekki et al. [41]	Independent	National research funds	“The authors declare no conflicts of interest.”	IQOS (THS 2.2)	HCI, puff volume, 55 mL; puff duration, 2 s; puff interval 30 s; ventilation blocking 100	1. Toxic gases (CO)<br>2. TSNAs	1. GC-FID and NDIR<br>2. LC-MS/MS
14.	Hoshino et al. [43]	Independent	Partly funded by Smoking Research Foundation.	”The authors declare no competing financial interest.”	ILUMA ONE	HCI, puff volume, 55 mL; puff duration, 2 s; puff interval 30 s; ventilation blocking 100	1. Carbonyls<br>2. VOCs<br>3. TSNAs	1. LC-MS/MS<br>2. GC-MS<br>3. LC-MS/MS
15.	Dusautoir et al. [45]	Independent	National research funds	“The authors declare that they have no conflict of interest with tobacco, HTP or e-cig industries.”	IQOS 2.4	HCI, puff volume, 55 mL; puff duration, 2 s; puff	1. Carbonyls<br>2. PAHs	1. HPLC (UHPLC System with UV/VIS Detector)

						interval 30 s; ventilation blocking 100.		2. HPLC
16.	Kärkelä et al. [46]	Independent	Funded by PMI	"The research reported was funded by Philip Morris International."	THS 2.2	HCI, puff volume, 55 mL; puff duration, 2 s; puff interval 30 s; ventilation blocking 100.	1. Carbonyls<br>2. Toxic gases (CO)	1, 2. FTIR
17.	Gunduz et al. [49]	PMI (IQOS manufacturer)	Funded by PMI	"All authors are employees of Philip Morris International."	THS 3.0, regular and menthol sticks	HCI, puff volume, 55 mL; puff duration, 2 s; puff interval 30 s; bell-shaped puff profile; ventilation blocking 100.	1. Carbonyls<br>2. Toxic gases (CO)<br>3. VOCs<br>4. TSNAs<br>5. PAHs<br>6. Phenolics<br>7. Toxic metals	1-7. Validated targeted analytical methods (Labstat International ULC)
18.	Gonzalez-Suarez et al. [52]	PMI (IQOS manufacturer)	Funded by PMI	"All authors are employees of Philip Morris International. Philip Morris International is the sole source of funding and sponsor of this project."	THS 2.2	HCI, puff volume, 55 mL; puff duration, 2 s; puff interval 30 s; ventilation blocking 100.	1. Carbonyls<br>2. Toxic gases (CO)<br>3. VOCs<br>4. TSNAs<br>5. PAHs<br>6. Phenolics<br>7. Toxic metals	1, 4. LC-MS (liquid chromatography–electrospray ionization tandem mass spectrometry)<br>2. nondispersive infrared photometry<br>3, 5, 6. GC-MS<br>7. ICP-MS
19.	Keyser et al. [53]	Authors affiliated with another tobacco producer (subsidiary of British	Not reported	"The authors declare the following financial interests/personal relationships which may be considered as potential competing interests: Brian M Keyser reports financial support was provided by Reynolds American Inc. I, RL, JW, KJ, KZ, and PM are full time	IQOS (not further specified)	HCI	Carbonyls	LC-MS

American Tobacco)	employees of RAI Services Company which is part of Reynolds American Inc. Brian M Keyser reports a relationship with Reynolds American Inc that includes: employment. Brian Keyser, Robert Leverette, John Wertman, Kristen Jordan, Ken Szeliga, and Patrudu Makena are full time employees of RAI Services Company (RAIS). Thomas Shutsky and Reagan McRae are former employees of RAIS and were full time employees during the conduct of these studies. RAIS is a wholly owned subsidiary of Reynolds American, Inc., which is a wholly owned subsidiary of British American Tobacco plc (BAT). The data presented in this manuscript were generated and analyzed in studies commissioned by RAI Services Company and conducted under contract at LabCorp Early Development Laboratories Ltd or Labstat International Inc. If there are other authors, they declare that they have no known competing financial interests or personal relationships that could have appeared to influence the work reported in this paper.”	

20.	Stabbert et al. [54]	PMI (IQOS manufacturer)	No funding declaration provided	Acknowldgment – "The authors are grateful to Lynda Conroy for critically reviewing the manu-script, to the staff at the York Manufacturing Facility and Ashland MachineryDevelopment Facility for the production of the EHC and the heaters and to thestaff at Philip Morris Research Laboratories for their excellent technical assist-ance. Furthermore, the authors would like to thank Dr Barbara Wiecken(Henkel KGaA, Düsseldorf) for the metal analyses."	Early generation product	35-ml puff of 2 s duration taken once every 60	1. Carbonyls<br>2. Toxic gases (CO)<br>3. VOCs<br>4. TSNAs<br>5. PAHs<br>6. Phenolics<br>7. Toxic metals	1. LC<br>2. nondispersive infrared photometry<br>3, 5, 6. GC-MS<br>4. GC<br>7. graphite furnace atomic absorption spectrometry
21.	Lim et al. [55]	Independent	National research funds	"The authors declare that they have no known competing financial interests or personal relationships that could have appeared to influence the work reported in this paper."	IQOS (IQOS)	10 puffs; 20 mL/ puff, 2 s puff duration; 30 s interval	1. VOCs	1. Thermal desorption - Gas chromatography - Mass spectrometry

Table S2. Additional publications used as supporting evidence but not included in the systematic synthesis.

No.	Ref.	Year	Institutional affiliation/ Funding	Purpose in the review
1.	Bitzer et al. [10]	2020	Independent/ national research funds	Presence of free radicals in THP aerosols compared with cigarette smoke, toxicological relevance
2.	Shein & Jeschke [11]	2019	Independent/ Funded by PMI	Presence of free radicals in THP aerosols compared with cigarette smoke, toxicological relevance, proposal of alternative analytical method - electron paramagnetic resonance
3.	Heide et al. [13]	2019	Independent / No funding declared	Proposal of puff regimen variation, alternative analytical method - single photon ionization time-of-flight mass spectrometry

4.	Schaller et al. [14]	2016	PMI (IQOS manufacturer) / Funded by PMI	Overall reduction in target compounds THP vs classic cigarettes; the authors used candidate products developed by the company, not commercially available products
5.	Davis et al. [19]	2019	Independent/ national research funds	Evaluation of polymeric film/components of filters degradation, presence of toxic compounds
6.	Tane et al. [22]	2024	Independent/ Funded by PMI	Evaluation of compounds at different working temperatures, proposal of a non-standardized working set-up
7.	Lang et al. [24]	2024	PMI (IQOS manufacturer) / Funded by PMI	Exclusive assement of compounds outside the proposed panel, analytical methodology, toxicological relevance
8.	Gasparyan et al. [27]	2018	Affiliated with another producer from tobacco industry (British American Tobacco)/ Funded by British American Tobacco	Exclusive assement of compounds outside the proposed panel, analytical methodology, toxicological relevance
9.	Pratte et al. [28]	2017	PMI (IQOS manufacturer) / Funded by PMI	Exclusive assement of compounds outside the proposed panel, analytical methodology
10.	Mottier et al. [29]	2016	PMI (IQOS manufacturer) / Funded by PMI	Analytical methodology, toxicological relevance
11.	Savareear et al. [38]	2018	Affiliated with another producer from tobacco industry (British American Tobacco)/ Funded by British American Tobacco	Analytical methodology (proposal of TD-GC × GC-(HR)TOFMS/FID), toxicological relevance
12.	Rodrigo et al. [40]	2021	PMI (IQOS manufacturer) / Funded by PMI	Analytical methodology, toxicological relevance
13.	McAdam et al. [42]	2019	Affiliated with another producer from tobacco industry (British American Tobacco)/ Funded by British American Tobacco	Analytical methodology, toxicological relevance
14.	Cerciello et al. [44]	2025	Independent/ Funded by PMI	Analytical methodology, toxicological relevance, overall VOCs and PAHs levels
15.	Lee et al. [47]	2018	Independent/ national research funds	Analytical methodology, toxicological relevance
16.	Wang et al. [48]	2021	Independent/ national research funds	Analytical methodology, toxicological relevance
17.	Ishikawa et al. [56]	2018	Authors affiliated with another tobacco producer (Japan Tobacco Inc.) / Funded by Japan Tobacco Inc.	Analytical methodology, toxicological relevance

18.	Enomoto et al. [57]	2022	Authors affiliated with another tobacco producer (Japan Tobacco Inc.) / Funded by Japan Tobacco Inc.	Analytical methodology, overall reduction of VOCs compared with conventional cigarettes
19.	Borgerding et al. [59]	1998	Affiliated with another producer from tobacco industry (R. J. Reynolds Tobacco Company)/ Funding not declared	Analytical methodology
20.	Eaton et al. [60]	2017	Authors affiliated with another tobacco producer (British American Tobacco) / Funded by British American Tobacco Investments Ltd	Analytical methodology, toxicological relevance
19.	Poynton et al. [61]	2017	Authors affiliated with another tobacco producer (British American Tobacco) / Funded by British American Tobacco Investments Ltd	Analytical methodology, toxicological relevance
20.	Hirn et al. [62]	2020	Authors affiliated with another tobacco producer (Japan Tobacco Inc.) / Funded by Japan Tobacco Inc.	Analytical methodology, toxicological relevance
21.	Forster et al. [63]	2018	Authors affiliated with another tobacco producer (British American Tobacco) / Funded by British American Tobacco Investments Ltd	Analytical methodology, toxicological relevance
22.	Ichitsubo et al. [64]	2018	Authors affiliated with another tobacco producer (Japan Tobacco Inc.) / Funded by Japan Tobacco Inc.	Analytical methodology, toxicological relevance
23.	Iskandar et al. [65]	2017	PMI (IQOS manufacturer) / Funded by PMI	Toxicological relevance
24.	Pacitto et al. [66]	2018	Independent / Funding not declared	Analytical methodology, overall reduction of volatile and non-volatile fractions compared with conventional cigarettes
25.	Gu et al. [67]	2025	Independent / national research funds	Analytical methodology, evalaution of aerosolo particle morphology

26.	Taylor et al. [68]	2017	Authors affiliated with another tobacco producer (British American Tobacco) / Funded by British American Tobacco Investments Ltd	Analytical methodology, toxicological relevance
27.	Farsalinos et al. [69]	2018	Independent / No funds received	Analytical methodology
28.	Xiao et al. [72]	2026	Independent / Funded by China Tobacco Guangxi Industrial Co.	Analytical methodology, toxicological relevance
