# Supplementary material for: Beyond the WHO Priority Toxicants: A Systematic Review of Harmful and Potentially Harmful Constituents in IQOS Aerosols
Source: Toxics. 2026 Jul 13;14(7):614. doi: 10.3390/toxics14070614 (PMC13417897; doi:10.3390/toxics14070614)
Supplement: Supplementary file 1 [file toxics-14-00614-s001.zip › Supplementary material.pdf]

Supplementary material

Beyond the WHO Priority Toxicants: A Systematic Review of Harmful and Potentially Harmful Constituents in IQOS Aerosols

Roxana Ioana Matei [1], Anda Maria Baroi [1], Toma Fistos [1], Irina Fierascu [1,2,†] and Radu Claudiu Fierascu [1,3,4,*,†]

[1] National Institute for Research & Development in Chemistry and Petrochemistry—ICECHIM Bucharest, 202 Splaiul Independenței, 060021 Bucharest, Romania; roxana.brazdis@icechim.ro (R.I.M.); anda.baroi@icechim.ro (A.M.B.); toma.fistos@icechim.ro (T.F.); irina.fierascu@icechim.ro (I.F.)
[2] Faculty of Horticulture, University of Agronomic Sciences and Veterinary Medicine of Bucharest, 59 Marasti Blvd, District 1, 011464 Bucharest, Romania
[3] Faculty of Chemical Engineering and Biotechnologies, National University of Science and Technology Politehnica Bucharest, 1-7 Gheorghe Polizu St., 011061 Bucharest, Romania
[4] Academy of Romanian Scientists, 3 Ilfov Str., 050044 Bucharest, Romania
* Correspondence: fierascu.radu@icechim.ro
† These authors contributed equally to this work.

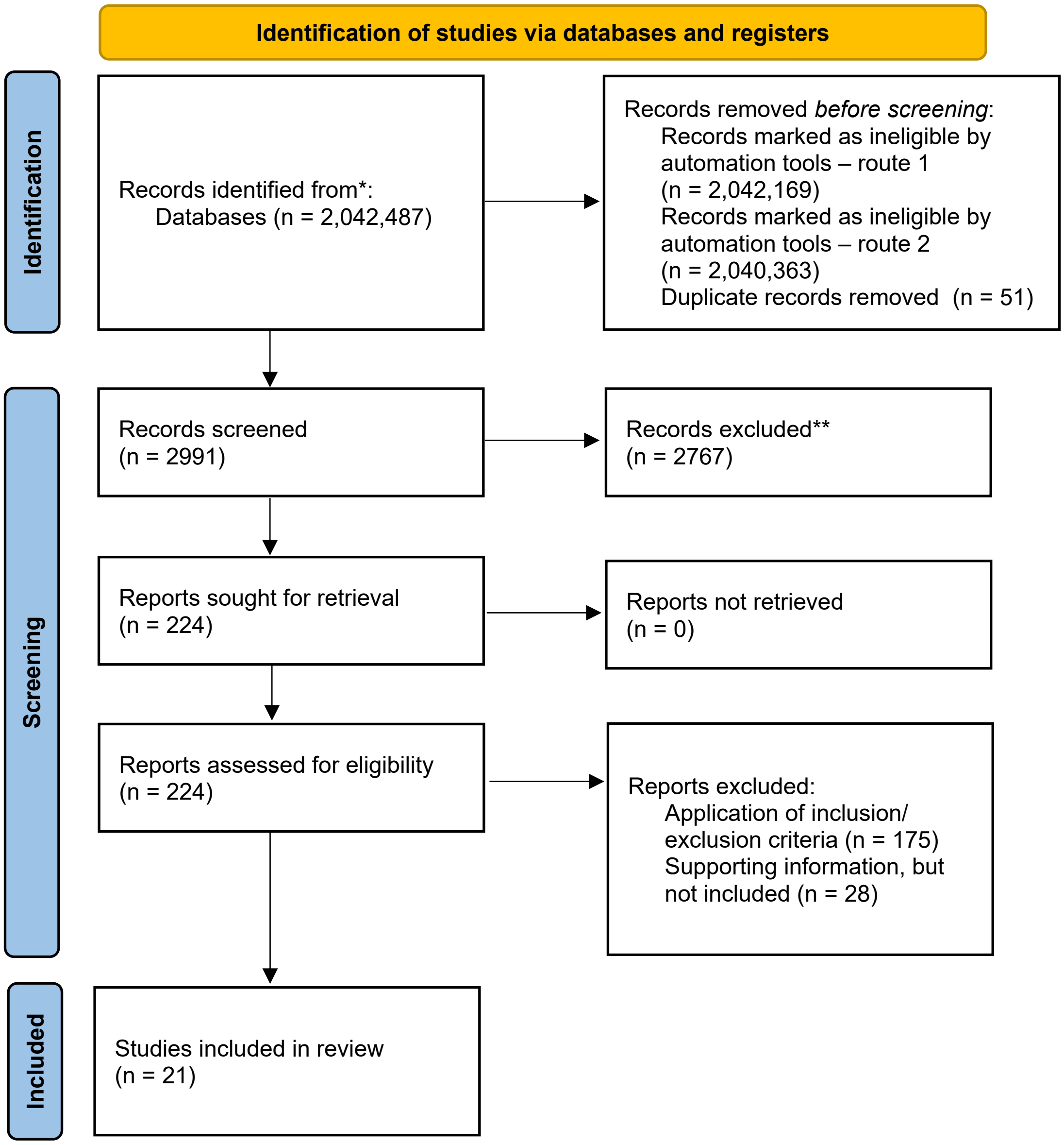

PRISMA 2020 flow diagram for new systematic reviews which included searches of databases and registers only

*Consider, if feasible to do so, reporting the number of records identified from each database or register searched (rather than the total number across all databases/registers).

**If automation tools were used, indicate how many records were excluded by a human and how many were excluded by automation tools.

Figure S1. PRISMA flow diagram.

Source: Page MJ, et al. BMJ 2021;372:n71. doi: 10.1136/bmj.n71.

This work is licensed under CC BY 4.0. To view a copy of this license, visit https://creativecommons.org/licenses/by/4.0/
